# Synchronous Primary Hyperparathyroidism and Polycythemia Vera: A Case Report and Literature Review

**DOI:** 10.1002/ccr3.71539

**Published:** 2025-11-29

**Authors:** Maryam Montazeri, Pooria Sobhanian, Seyed Mohammad Sakhaei, Homina Saffar

**Affiliations:** ^1^ Razi Clinical Researches Development Mazandaran University of Medical Science Sari Iran; ^2^ Faculty of Medicine Mazandaran University of Medical Sciences Sari Iran; ^3^ Department of Radiology, School of Medicine Mazandaran University of Medical Sciences Sari Iran

**Keywords:** parathyroid adenoma, parathyroidectomy, polycythemia vera, primary hyperparathyroidism

## Abstract

Polycythemia vera (PV) is a chronic myeloproliferative neoplasm characterized by increased erythrocytes and commonly associated with JAK2 mutations. Primary hyperparathyroidism (PHPT), which is often caused by a parathyroid adenoma, is a common cause of hypercalcemia. Although unrelated, a potential association between PV and PHPT has been described in the literature. We report the case of a 41 year‐old male presenting with concurrent PV and PHPT due to a parathyroid adenoma. Following parathyroidectomy, the patient's hemoglobin and hematocrit levels normalized without further treatment, suggesting remission of PV. This case report and literature review highlight a possible relationship between the calcium–parathyroid hormone axis and hematopoiesis, providing insight into potential shared pathophysiological mechanisms.

**Summary**

- Concurrent polycythemia vera and primary hyperparathyroidism are rare but clinically significant; parathyroidectomy may lead to hematologic remission, highlighting the possible relationship between the two diseases.

## 1 Introduction

Polycythemia vera (PV) is a JAK2‐mutated myeloproliferative neoplasm characterized by clonal erythrocytosis [1, 2]. Concurrent primary hyperparathyroidism (PHPT) is extremely rare; to the best of our knowledge, only 10 adult cases have been reported in the English literature since 1981 (Table 1).

**TABLE 1:** A Review of Studies on Adults with PV and PHPT and Patient Characteristics.

Study	Gender/Age	Pre‐op laboratory parameters	Sign & symptom	Radiologic finding	Management	PHPT outcome	PV outcome (post‐parathyroidectomy)
Godeau et al. 1981 [3]	Male, 54	Hb 23 g/dL, Hct 65%, Ca 14.0 mg/dL, PTH 1.28 ng/mL	Left arm paralysis, Left facial paralysis, Dysphonia, Obtundation, Left‐sided hemiparesis (upper extremity predominant), Left Babinski sign	Ischemic region in the right parietorolandic area, with no evidence of tumor on cerebral vessel angiography.	Mithramycin, Demeclocycline, Parathyroidectomy	Drop in serum Ca after surgery (10.5 mg/dL)	Resolved—Normalized Hb (12.0 g/dL) and Hct (39%)
Boivin et al. 1992 [4]	Male, 71	RBC count in the range of 5.5–5.7 × 10^12^/l, Ca 2.80 mmol/L	Recurrent superficial thrombophlebitis of the lower limbs	Ectopic parathyroid gland located on the left side, situated between the inferior cervical segment of the esophagus and the proximal portion of the left internal carotid artery, as identified on CT scan.	Exploratory surgery	Not reported	Not reported
	Male, 58	Hb 18.1 g/dL, Hct 53.2%, Ca 2.6 mmol/L	Severe asthenia, hyperalgesia	Not mentioned	Furosemide, mithramycin, Calsyn	Persistent hypercalcemia secondary to parathyroid adenoma (4.5 mmol/L)	Not resolved—patient died from complications of hypercalcemia
	Male, 81	Hb 19.0 g/dL, Hct 57%, Ca 2.9 mmol/L, PTH 214 μmol/ml	Venous thrombosis of the right lower extremity	No parathyroid adenoma detected on CT scan	Not mentioned	Not reported	Not reported
Silverberg et al. 2005 [5]	Male, 58	Hct 55%, Ca 10.2 mg/dL, ionized Ca 1.37 mmol/L, intact PTH 71 pg/mL	Asymptomatic	Parathyroid adenoma in the left superior mediastinum approximately 4–5 cm inferior to the left thyroid lobe on TcMIBI scan	Parathyroidectomy, Phlebotomy	Drop in serum Ca and PTH	Resolved—Decrease in Hb and Hct (43.7%), symptoms improved
Bae et al. 2012 [6]	Female, 59	Hb 18.2 g/dL, Hct 55.1%, Ca 12.6 mg/dL, PTH 221 pg/mL	Hypercalcemia	Nodular lesion left parathyroid on ultrasonography and scintigraphy	Parathyroidectomy	Drop in serum Ca (9.8 mg/dL) and PTH (34 pg/mL)	Resolved—Decrease in Hb (15.5 g/dL) and Hct (46.7%), symptoms improved
Kulaylat et al. 2014 [7]	Female, 84	Median Hb 13.4 g/dL, Ca 10.6 mg/dL, PTH 70 pg/mL, Negative JAK‐2 V617F	Constipation, musculoskeletal pain, and concentration difficulties	Osteoporosis on bone densitometry, left lower parathyroid adenoma on TcMIBI scan and ultrasound	Parathyroidectomy, Hydroxyurea, Phlebotomy	Normal Ca (9.1 mg/dL) and PTH (28 pg/mL)	Partially resolved—Transient drop in Hb (10.5 g/dL) and Hct level
Abdalhadi et al. 2020 [8]	Female, 64	Hb 12.8 g/dL, Hct 37.3%, 2.73 mmol/L, corrected Ca 2.87 mmol/L, PTH 183 pg/mL, Positive JAK2 V617F	Fatigue, generalized bone pain, constipation	Bilateral parathyroid nodules detected on ultrasound; severe osteoporosis confirmed by DEXA scan; Tc‐99 m MIBI scan negative for enlarged or hyperactive parathyroid tissue; parathyroid SPECT–CT revealed a hyperfunctioning parathyroid adenoma in the left upper thyroid pole region.	Parathyroidectomy, Cinacalcet	Not reported	Not reported
Padmakumar et al. 2020 [9]	Female, 52	Hb 18.8 g/dL, Ca 13.53 mg/dL, PTH 569.9 pg/mL, Positive JAK‐2	Fever, headache, generalized weakness	Left inferior parathyroid adenoma on TcMIBI scan and ultrasound	Parathyroidectomy, Phlebotomy	Drop in serum Ca (9.1 mg/dL) and PTH (28 pg/mL)	Resolved—Decrease in Hb (14.3 g/dL) and Hct, symptoms improved
Sthaneshwar et al. 2022 [10]	Female, 59	Hb 15.8 g/dL g/dL, Hct of 71%, Ca 2.9 mmol/L, PTH 11.6 pmol/L, positive JAK2 V617F mutation	Headaches, polyuria, constipation	Left upper‐pole parathyroid adenoma on TcMIBI scan and ultrasound	Parathyroidectomy, Hydroxyurea, Phlebotomy	Normal Ca (2.6 mmol/L) and PTH (5.3 pmol/L)	Resolved—Decrease in Hb (14.7 g/dL) and Hct (44%), reduction in the need for hydroxyurea consumption, no need for further phlebotomy

Abbreviations: Ca, calcium; DEXA scan, dual‐energy X‐ray absorptiometry; Hb, hemoglobin; Hct, hematocrit; PHPT, primary hyperparathyroidism; PTH, parathyroid hormone; PV, polycythemia vera; RBC, red blood cells; TcMIBI, Technetium 99 M Sestamibi Scan.

Several patients showed complete or partial hematologic remission following parathyroidectomy, suggesting a possible pathophysiologic link [7, 8, 9, 11, 12]. PHPT accounts for approximately 90% of hypercalcemia cases in outpatient settings [13].

We describe the eleventh documented case of concomitant PV and PHPT with sustained remission of erythrocytosis 12 months after parathyroidectomy.

## 2 Case History

A 41 year‐old male presented to the clinic with a history of recurrent kidney stones for a year and complaints of headache and generalized weakness. The patient had no significant past medical history except for recurrent nephrolithiasis over the previous year, which led to further evaluation. There was no known family history of hyperparathyroidism, PV, or related hematologic or endocrine conditions. The patient was a nonsmoker with no history of tobacco use. Residence was in a low altitude region, ruling out hypoxia‐driven secondary erythrocytosis due to high altitude. The patient's physical examination revealed no significant findings, except for a plethoric face.

## 3 Differential Diagnosis, Investigations, and Treatment

Laboratory investigations revealed a total serum calcium of 11.6 mg/dL (8.5–10.5), serum albumin 5.06 g/dL, corrected calcium 10.75 mg/dL, 24 h urine calcium was normal, and an intact PTH 124 pg/mL (10–65). Complete blood count (CBC) showed a hemoglobin level of 17.9 g/dL (13.5–17.5) and a hematocrit level of 50.0% (37–53). The patient underwent hematological and endocrinological workup according to the abnormal tests.

Bone marrow aspiration and biopsy revealed a mildly hypercellular marrow (60%–70%) with prominent erythroid hyperplasia and a mild left shift in the myeloid series. Megakaryocytes were adequate in number, morphologically unremarkable, with no significant atypia, clustering, or dysplasia. There was no evidence of trilineage proliferation (panmyelosis). These findings indicate predominant erythroid proliferation rather than the classic panmyelosis typically seen in primary PV. However, when correlated with the presence of JAK2 V617F mutation, subnormal erythropoietin level (0.3 mIU/mL, normal 3.5–26.0), and persistent erythrocytosis, the overall picture fulfilled the 2016 WHO diagnostic criteria for PV (major criteria 1 + 2 + 3, plus minor criterion) despite the absence of panmyelosis on this single biopsy [14].

**FIGURE 1:**
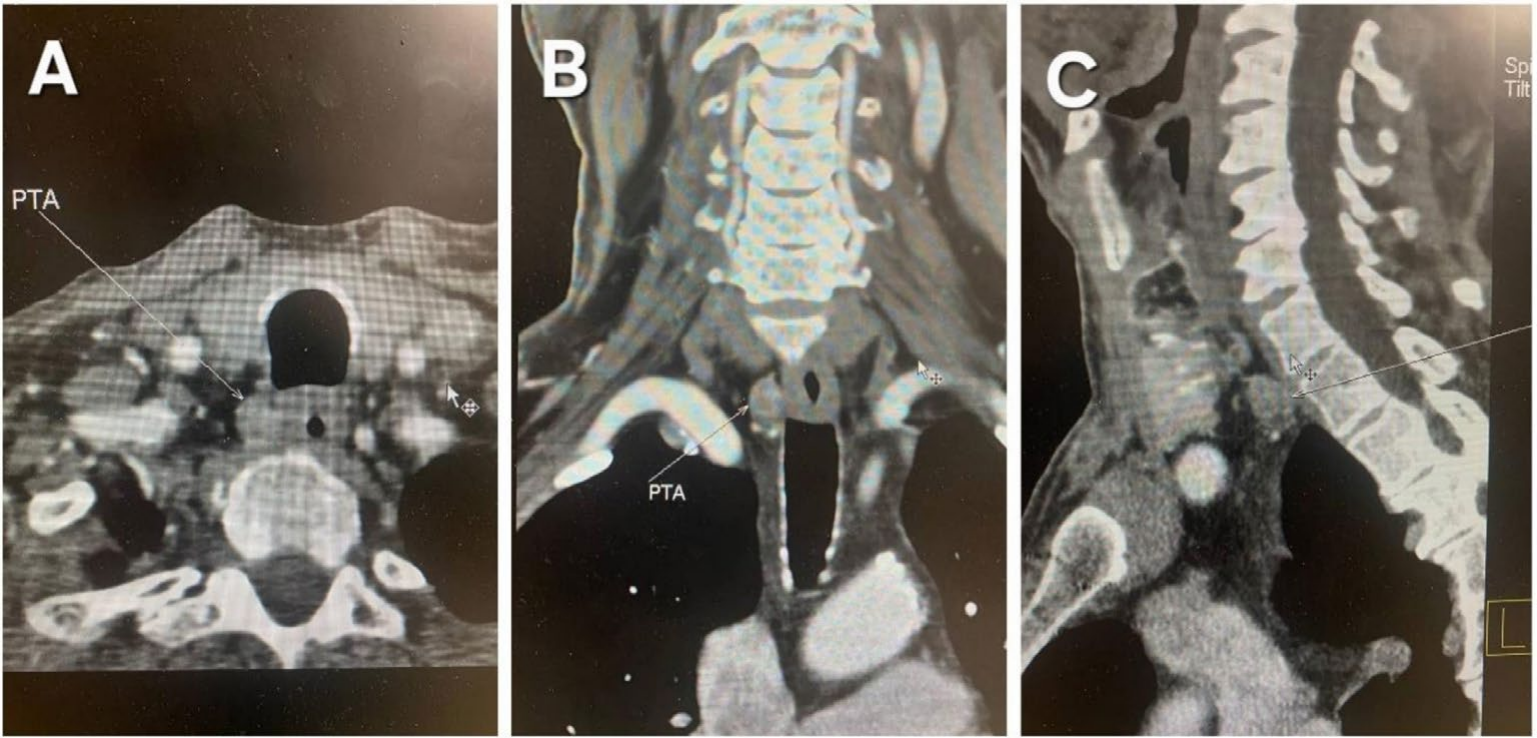
Neck CT scan with contrast study in axial (A), coronal (B), and sagittal (C) views: A 24 × 14 mm hyperenhancing, well‐circumscribed, ovoid nodule in the inferior right retrothyroid region of the right paratracheal space, consistent with parathyroid adenoma.

A neck ultrasonography detected a hypoechoic nodule in the right retrothyroid region, suggestive of a parathyroid adenoma. There was no evidence of cervical lymphadenopathy. The dual‐phase Technetium 99 M Sestamibi Scan (TcMIBI) was negative for parathyroid adenoma. However, a parathyroid scan with SPECT–CT showed a 15 mm parathyroid adenoma posterior to the right thyroid lobe (Figure 1).

The patient was referred to the surgical team to proceed with parathyroidectomy due to the diagnosis of symptomatic PHPT secondary to a parathyroid adenoma. Given the paradoxically increased perioperative risk of both thrombosis and hemorrhage in PV due to erythrocytosis and hyperviscosity, preoperative DVT prophylaxis followed standard protocols with enoxaparin 40,000 IU (equivalent to 40 mg) subcutaneously once daily, starting 12–24 h before surgery and continued postoperatively until full mobilization. Graduated compression stockings provided mechanical prophylaxis. Hematocrit remained below 45%, and low‐dose aspirin continued perioperatively due to low bleeding risk [15, 16]. A hematologist participated in preoperative planning to optimize counts and balance thrombotic/bleeding risks. Histopathological assessment confirmed the diagnosis of parathyroid adenoma.

## 4 Outcome and Follow‐Up

**FIGURE 2:**
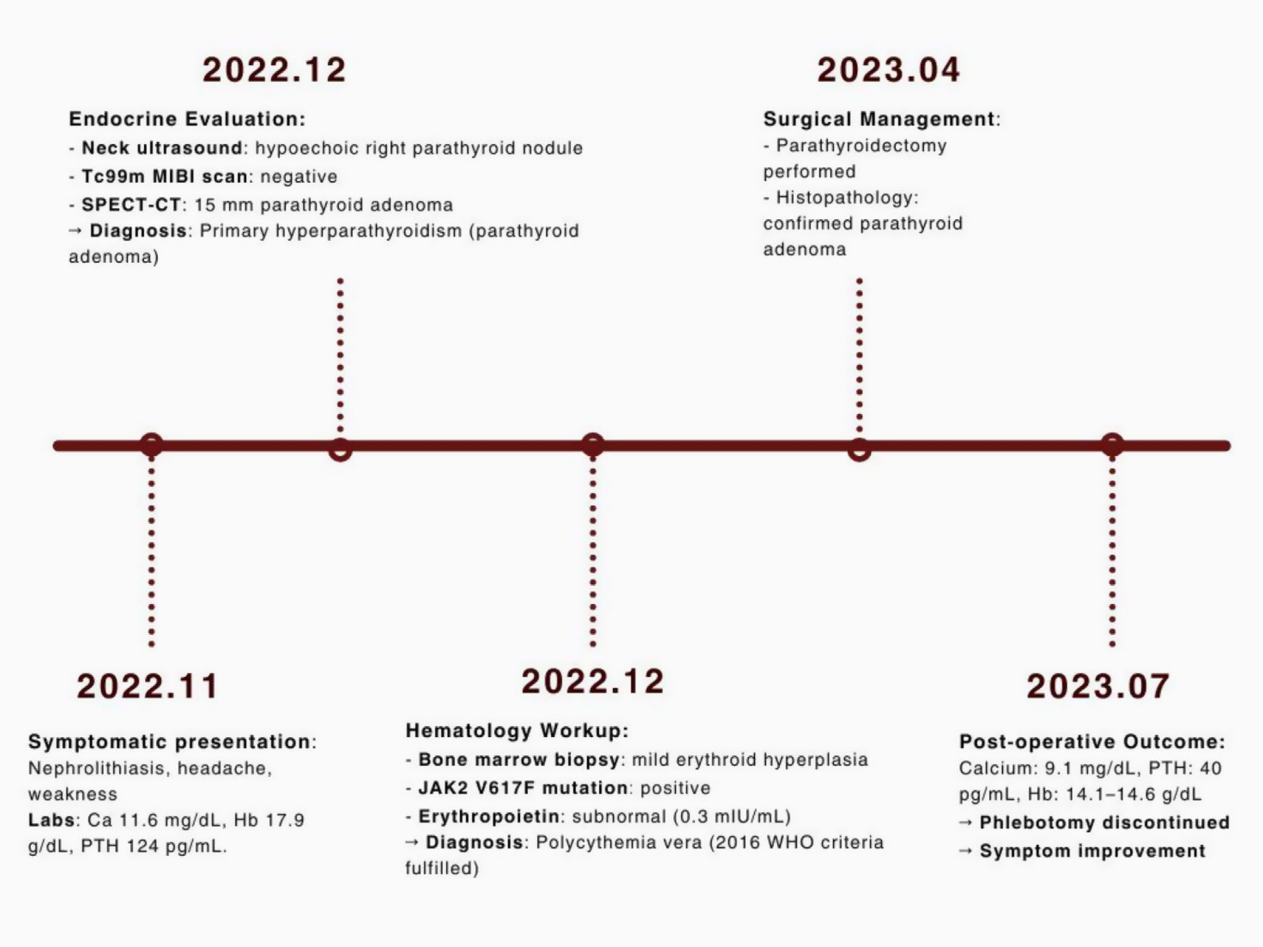
Timeline of case presentation, diagnosis, treatment, and follow‐up.

In subsequent postoperative visits, the patient exhibited hemoglobin levels of 14.1 g/dL and 14.6 g/dL, hematocrit levels of 42.1% and 44%, a calcium level of 9.1 mg/dL, and a PTH level of 40 pg./mL. As the hemoglobin and hematocrit levels normalized, phlebotomy was discontinued for the patient. The patient also noted a subjective improvement in his primary symptoms (Figure 2).

## 5 Discussion

PV is a subtype of CMPNs characterized by clonal multipotent stem cell disease, distinguished by an increased production of erythrocytes relative to other blood cells and a positive JAK2 mutation in > 95% of patients [1, 2]. The 2016 WHO diagnostic criteria for PV include three major criteria and one minor criterion: hemoglobin > 16.5 g/dL and > 16.0 g/dL in men and women, respectively, or hematocrit > 49% (men)/> 48% (women), or an increased red cell mass; bone marrow biopsy showing hypercellularity for age with trilineage growth (panmyelosis) including prominent erythroid, granulocytic, and megakaryocytic proliferation with pleomorphic, mature megakaryocytes (differences in size); and the presence of JAK2 or JAK2 exon 12 mutation. A subnormal serum erythropoietin level is the minor criterion. For a PV diagnosis, either all major criteria are required or the first two major criteria plus a subnormal serum erythropoietin level [14]. Our patient met all diagnostic criteria for PV.

Although classic PV is characterized by trilineage proliferation (panmyelosis), several JAK2‐positive cases with isolated erythroid hyperplasia have been reported, particularly in the context of secondary stimuli such as hyperparathyroidism‐induced hypercalcemia [11, 17]. The absence of panmyelosis in our patient does not preclude the diagnosis when other WHO criteria are unequivocally met.

Hypercalcemia in CMPNs such as PV often occurs following malignancies such as renal cell carcinoma and is rarely associated with CMPN complications [6, 8, 9]. Cases of hypercalcemia following PHPT in PV patients have been reported in the literature (Table 1). This association was first reported by Berlin et al. in 1949 [12]. Fallah et al. reported a twofold increased risk of PV following parathyroid adenoma (men fivefold) and an increased risk of parathyroid adenoma following PV (men about eightfold; women threefold) [18]. The precise biological mechanisms linking PV and PHPT are not known, but studies suggest a relationship between major calcium‐regulating hormones such as PTH, osteoclastic/osteoblastic activity, and hematopoiesis regulation. Parathyroid tumors may induce pancytosis through the production of a growth factor, especially in the presence of ionized hypercalcemia. Additionally, the calcium–PTH axis can directly impact hematopoietic precursors and contribute to erythropoiesis activation [7, 9, 10, 19]. PTH has been shown to enhance Fe 59 incorporation into erythrocytes of polycythemic mice and regulate normal erythropoiesis [20]. Considering the higher risk of PV following parathyroid adenoma in men compared to women, it seems that gender‐specific hormonal or genetic alterations (e.g., X‐linked with recessive inheritance) are also possible mechanisms of this relationship [18].

In our case, the initial planar Tc‐99 m MIBI scan was negative, while SPECT–CT revealed a 15 mm parathyroid adenoma posterior to the right thyroid lobe. This discrepancy can be attributed to the superior sensitivity of SPECT–CT over planar imaging. Planar scans may yield false‐negative results for small or posteriorly located adenomas due to limited depth resolution and overlapping structures (e.g., thyroid tissue), with reported sensitivity around 34%–56%. In contrast, SPECT–CT provides three‐dimensional functional imaging combined with anatomical detail, improving detection rates to approximately 62%–73% [21].

The standard criteria for parathyroidectomy following PHPT involve the presence of symptoms related to hypercalcemia and hyperparathyroidism. In asymptomatic patients, surgery is determined by measurable laboratory abnormalities consistent with consensus statements for intervention (e.g., serum calcium 1.0 mg/dL above normal, reduced creatinine clearance) [7, 22]. In our patient, due to the presence of symptoms such as nephrolithiasis and headache, parathyroidectomy was performed, and the patient's symptoms improved postsurgery. Several cases have described the remission or improvement of PV following parathyroidectomy in the context of concomitant disease, and in our patient, remission of PV was observed without any treatment [3, 5, 6, 9, 10]. Padmakumar et al. reported a patient with PV who had elevated PTH levels during periodic testing, which was found on further workup for a parathyroid adenoma. Two weeks after parathyroidectomy, the patient's hemoglobin levels normalized without the need for medication or venesection, and it was suggested that all PV patients be screened for hyperparathyroidism [9]. Weinstein et al. reported that the reduction of calcium levels in the postoperative period is associated with the remission of PV. However, the recurrence of hypercalcemia in their patient resulted in pancytosis [23]. Kulaylat et al. demonstrated a transient improvement of hemoglobin levels in a JAK2‐negative patient after parathyroidectomy and stated that in cases with a close temporal relationship between the development of hyperparathyroidism and PV, remission of PV can be expected after parathyroid surgery [7].

Perioperative management in concomitant PV and PHPT faces challenges from dual thrombosis (hyperviscosity/erythrocytosis) and hemorrhage risks. Guidelines recommend antithrombotic prophylaxis per standard protocols (typically low‐molecular‐weight heparin such as enoxaparin 40 mg/40,000 IU daily, plus mechanical methods like graduated compression stockings), strict hematocrit control below 45%, aspirin continuation in low‐bleeding‐risk cases, and hematologist involvement [15, 16]. Our multidisciplinary approach prevented thrombotic/hemorrhagic events, consistent with British Society for Hematology (Grade 1B) and current expert recommendations.

Our case further supports the relationship between PV and PHPT and documents remission of PV following parathyroidectomy, consistent with other studies. However, while hemoglobin and hematocrit levels normalized post‐parathyroidectomy, this cannot be definitively attributed to complete remission of PV, as the causal link between PHPT and PV remains unclear in the literature, and other factors (e.g., resolution of hypercalcemia) may have contributed [7, 9, 23]. No follow‐up bone marrow biopsy or JAK2 allele burden assessment was performed due to the patient's clinical stability and sustained normalization of hematologic parameters; this represents a limitation of our case report. Future studies with long‐term follow‐up, including serial bone marrow evaluations and JAK2 monitoring, are recommended to better characterize the potential for true remission in such cases. Given the unclear nature of this relationship and the lack of well‐characterized long‐term outcomes, it is recommended to conduct studies on a larger number of patients with a longer follow‐up period.

## 6 Conclusion

The relationship between PV and hyperparathyroidism has been documented in the literature. Our case contributes additional support to the clinical relevance of this association by demonstrating the amelioration of PV symptoms following the surgical excision of a parathyroid adenoma. Further investigations are needed to elucidate the underlying mechanisms involving the calcium–PTH axis and its impact on erythropoiesis. Through emphasizing this uncommon observation, our study seeks to raise awareness among healthcare professionals in this specialty and enhance the care of patients with these conditions.

## Author Contributions

**Maryam Montazeri:** conceptualization, writing – review and editing. **Pooria Sobhanian:** data curation, writing – original draft. **Seyed Mohammad Sakhaei:** supervision, writing – review and editing. **Homina Saffar:** data curation, writing – original draft.

## Funding

The authors received no specific funding for this work.

## Ethics Statement

There were no ethical considerations to be considered in this research.

## Consent

The written informed consent for publication was obtained from the patient.

## Conflicts of Interest

The authors declare no conflicts of interest.

## Data Availability

The data of this article will be shared on request.
